# Decoding sTREM2: its impact on Alzheimer’s disease – a comprehensive review of mechanisms and implications

**DOI:** 10.3389/fnagi.2024.1420731

**Published:** 2024-06-07

**Authors:** Cui Lin, Yu Kong, Qian Chen, Jixiang Zeng, Xiaojin Pan, Jifei Miao

**Affiliations:** ^1^Shenzhen Bao’an District Hospital of Traditional Chinese Medicine, Shenzhen, Guangdong, China; ^2^Shenzhen Bao’an Traditional Chinese Medicine Hospital, Guangzhou University of Chinese Medicine, Shenzhen, Guangdong, China

**Keywords:** sTREM2, Alzheimer’s disease, neuroinflammation, microglial activation, TREM2

## Abstract

Soluble Triggering Receptor Expressed on Myeloid Cells 2 (sTREM2) plays a crucial role in the pathogenesis of Alzheimer’s disease (AD). This review comprehensively examines sTREM2’s involvement in AD, focusing on its regulatory functions in microglial responses, neuroinflammation, and interactions with key pathological processes. We discuss the dynamic changes in sTREM2 levels in cerebrospinal fluid and plasma throughout AD progression, highlighting its potential as a therapeutic target. Furthermore, we explore the impact of genetic variants on sTREM2 expression and its interplay with other AD risk genes. The evidence presented in this review suggests that modulating sTREM2 activity could influence AD trajectory, making it a promising avenue for future research and drug development. By providing a holistic understanding of sTREM2’s multifaceted role in AD, this review aims to guide future studies and inspire novel therapeutic strategies.

## 1 Introduction: unveiling the role of sTREM2 in Alzheimer’s disease

Alzheimer’s disease (AD), as the predominant cause of dementia globally, is delineated by the progressive deposition of amyloid-beta (Aβ) plaques and neurofibrillary tangles composed of hyperphosphorylated tau protein within the cerebral cortex (Holtzman et al., 2011). Despite considerable scientific endeavors, the multifaceted etiology and pathogenesis of AD continue to be inadequately elucidated, thus stymying the advancement of efficacious therapeutic modalities. Recent discourse has increasingly recognized the integral role of neuroinflammation and innate immune responses in the progression of AD, with particular focus on microglia—the CNS’s inherent immune constituents—as pivotal in the disease’s initiation and development (Hansen et al., 2018; Wang and Colonna, 2019). Microglia, constituting 5–10% of total brain cells, are essential in sustaining CNS homeostasis by monitoring brain parenchyma, phagocytizing cellular debris and apoptotic cells, and modulating synaptic plasticity (Deczkowska et al., 2020). In AD, microglia accumulate around Aβ plaques and demonstrate diverse activation phenotypes, ranging from pro-inflammatory states that potentially aggravate neuronal degeneration to protective states facilitating Aβ clearance and tissue restitution (Keren-Shaul et al., 2017; Deczkowska et al., 2020). The regulation of these heterogeneous microglial responses is partly governed by various cell surface receptors, including the triggering receptor expressed on myeloid cells 2 (TREM2).

TREM2, an innate immune receptor predominantly expressed on microglia within the CNS, mediates its signaling through the adaptor protein DAP12 (Klesney-Tait et al., 2006; Ulland et al., 2017). Interaction with ligands such as anionic lipids, lipoproteins, and Aβ instigates intracellular signaling cascades that enhance microglial survival, proliferation, chemotaxis, and phagocytic activity (Keren-Shaul et al., 2017; Zhao et al., 2018; Deczkowska et al., 2020). The importance of TREM2 in AD pathology is underscored by the discovery of heterozygous TREM2 mutations, such as R47H, which significantly increase the risk of developing late-onset AD (Guerreiro et al., 2013; Jonsson et al., 2013). These mutations are posited to diminish the receptor’s protective functions, especially concerning Aβ plaque management (Condello et al., 2015; Yuan et al., 2016). Notably, a soluble variant of TREM2 (sTREM2) detected in cerebrospinal fluid (CSF) and plasma exhibits dynamic concentration changes through the AD continuum, with initial elevations during early symptomatic phases followed by reductions in later stages (Piccio et al., 2008, 2016; Heslegrave et al., 2016; Suárez-Calvet et al., 2016a,b; Zhou W. et al., 2023; Yin T. et al., 2024). Elevated CSF sTREM2 levels correlate with attenuated cognitive decline and reduced cerebral atrophy in AD subjects (Ewers et al., 2019), proposing sTREM2 as a potential biomarker for microglial activation and a moderator of disease trajectory (Gu et al., 2023). Recent findings further suggest an association between minimal depressive symptoms in prodromal AD and amyloid pathology, mediated in part by alterations in CSF sTREM2 levels (Wang et al., 2022b). The accruing evidence underscores the potential of sTREM2 as both a biomarker and a therapeutic target in AD (La Rosa et al., 2023). This review aims to furnish a comprehensive understanding of sTREM2 within the AD framework, exploring its biogenesis, functional roles, and clinical implications, thus underlining the imperative for additional research to delineate its precise function in AD pathogenesis.

## 2 Biogenesis of sTREM2: mechanisms and variants

**Figure 1:**
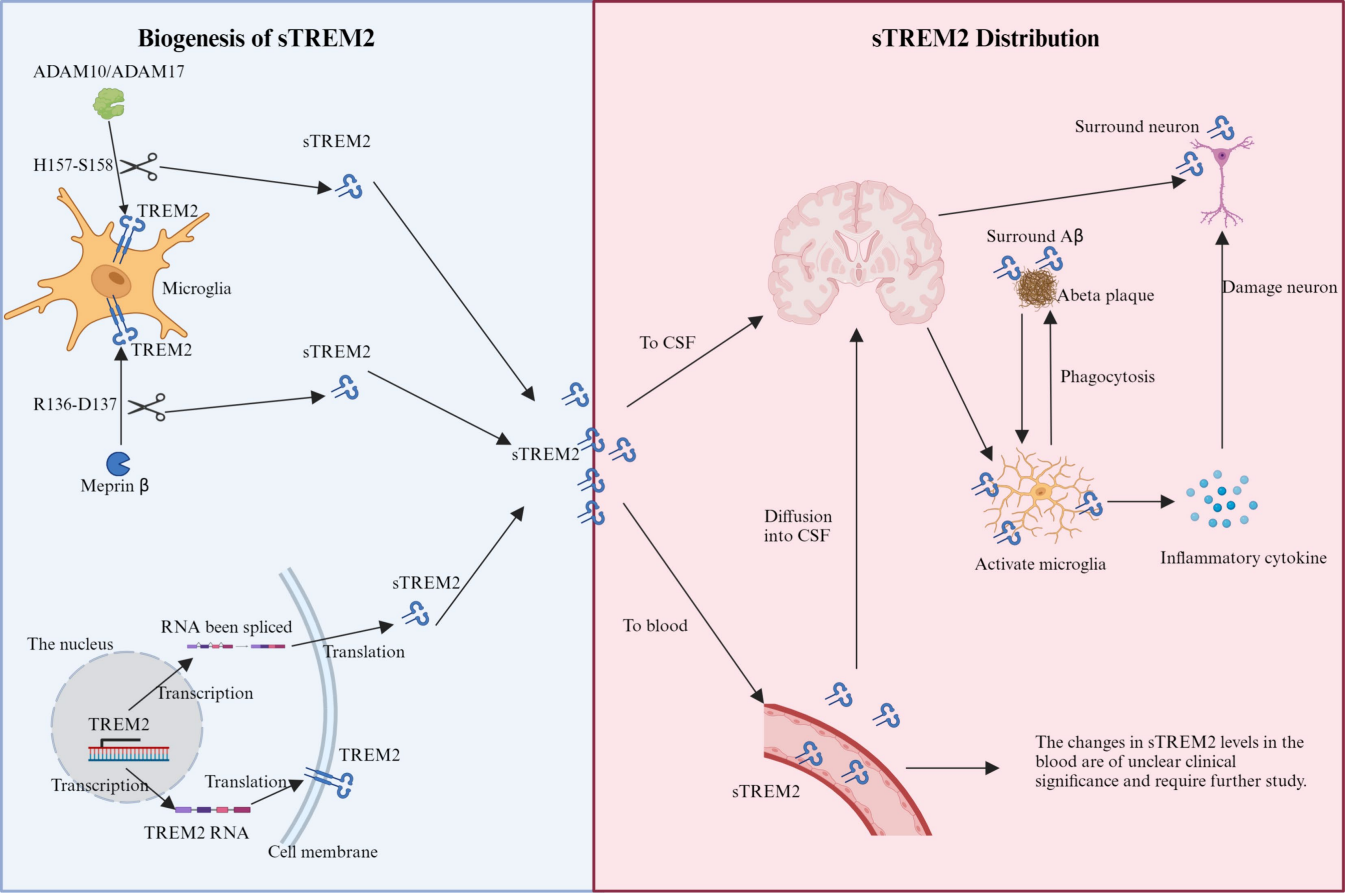
Schematic representation of the production and distribution of sTREM2. Biogenesis of sTREM2: the diagram illustrates two primary mechanisms of sTREM2 production within microglia. The proteolytic cleavage pathway involves the cleavage of the TREM2 ectodomain by metalloproteases ADAM10 and ADAM17 at the H157-S158 bond. Additionally, meprin β cleaves at the R136-D137 site. The splicing pathway shows mRNA variants being processed to produce sTREM2 lacking the transmembrane domain, which is then released into the extracellular space. sTREM2 distribution: sTREM2 is depicted as being distributed into two main compartments: the brain and the bloodstream. In the brain, sTREM2 penetrates the blood–brain barrier and accumulates around damaged neurons and amyloid-beta plaques, potentially exacerbating inflammatory responses. In the bloodstream, sTREM2 is shown being released by monocytes, illustrating its presence across different extracellular compartments.

sTREM2 is produced through two primary mechanisms (Figure 1): proteolytic cleavage of the TREM2 ectodomain and alternative splicing of the TREM2 gene. Metalloproteases ADAM10 and ADAM17 facilitate the cleavage at the H157-S158 bond, releasing sTREM2 into the extracellular space (Schlepckow et al., 2017; Thornton et al., 2017). Meprin β is a zinc metalloproteinase that belongs to the astacin family of proteases. It is known to cleave a wide range of substrates, including extracellular matrix proteins, cytokines, and cell surface proteins. In the context of sTREM2 biogenesis, meprin β has been shown to cleave TREM2 at a distinct site (R136-D137) compared to the cleavage site of ADAM10 and ADAM17 (H157-S158). This alternative cleavage by meprin β contributes to the generation of sTREM2 in macrophages (Berner et al., 2020). Alternatively, splicing variations produce mRNA lacking the transmembrane domain, which translates into sTREM2 and is subsequently released (Ma et al., 2016; Del-Aguila et al., 2019). Around 25% of the brain’s total sTREM2 is produced through this alternative splicing pathway (Del-Aguila et al., 2019). A recent genome-wide association study (GWAS) identified four loci correlating with CSF sTREM2 levels, including the well-known *MS4A* gene cluster on chromosome 11 and three novel loci: *TREM2* on chromosome 6, *TGFBR2/RBMS3* on chromosome 3, and *NECTIN2/APOE* on chromosome 19 (Wang et al., 2024).

Four principal *TREM2* mRNA variants have been identified: ENST00000373113, ENST00000373122, ENST00000338469, and TREM2^Δe2^ (Jin et al., 2014; Kiianitsa et al., 2021). The canonical ENST00000373113 encodes the full-length TREM2 protein, comprising 230 amino acids across 5 exons. ENST00000373122, lacking exon 5, diverges in its sequence, initiating from an alternate start within exon 4, yielding a 222 amino acid variant. ENST00000338469, lacking the critical exon 4, likely results in a secretory form of TREM2 of 219 amino acids. TREM2^Δe2^, devoid of exon 2 which encodes the ligand-binding domain, might produce a non-functional receptor variant tethered to the membrane (Kiianitsa et al., 2021). Mutations within the TREM2 gene notably impact sTREM2 production, with certain variants leading to protein misfolding and retention in the endoplasmic reticulum, thereby reducing sTREM2 secretion (Kleinberger et al., 2014; Park et al., 2015; Song et al., 2017). Specific mutations in the receptor’s Ig-like domain, such as p.T66M and p.Y38C, lead to misfolding or retention, thus decreasing its cell surface expression and subsequent cleavage into sTREM2 and C-terminal fragments *in vitro* (Kleinberger et al., 2014; Park et al., 2015; Song et al., 2017). Conversely, variants like p.R47H do not affect intracellular trafficking (Kleinberger et al., 2014; Wang et al., 2015). Additionally, certain low-frequency genetic alterations in the *TREM2* gene could alter splicing regulatory elements, potentially increasing the probability of exon 2 omission during splicing (Han et al., 2021). Thus, a spectrum of genetic variations influences both the cleavage and splicing of TREM2, affecting the observed levels of sTREM2.

## 3 sTREM2 distribution: cellular sources and pathophysiological implications

sTREM2 exhibits a varied distribution within the extracellular domain, significantly influenced by its cellular origin. It is secreted into the brain’s parenchyma by microglia (Henjum et al., 2016; Tan et al., 2017) and released into the bloodstream by monocytes (Feuerbach et al., 2017; Figure 1), illustrating its presence across different extracellular spaces. This variability in secretion highlights sTREM2’s involvement in a spectrum of functions, particularly evident during the early stages of AD, where its accumulation around damaged neurons and protein plaques suggests a role in initiating innate immune responses and potentially exacerbating the disease state (Kober and Brett, 2017; Song et al., 2018). Unregulated proteolytic cleavage, which results in increased sTREM2 levels, can have significant consequences, such as the potential disruption of the blood–brain barrier and the diffusion of sTREM2 into the CSF (Raha-Chowdhury et al., 2019; Tanaka et al., 2019). Additionally, minor quantities of sTREM2 found in the plasma may infiltrate through compromised vascular structures into the brain parenchyma (Pugazhenthi et al., 2017), offering a partial explanation for the increased sTREM2 concentrations observed in AD alongside other neurodegenerative diseases like multiple sclerosis (MS), frontal temporal dementia (FTD), and Lewy body dementia (LBD) (Piccio et al., 2008; Cooper-Knock et al., 2017; Zhou et al., 2019).

The dynamic progression of sTREM2 levels in the CSF throughout the AD continuum, particularly their elevation during early disease stages, is crucial for both diagnostic and staging purposes (Song et al., 2018; Rauchmann et al., 2019). Certain studies have reported a rise in sTREM2 levels in patients up to 5 years before the clinical onset of AD (Suárez-Calvet et al., 2016a,b), where these elevated levels also correlate with established neurodegenerative markers such as t-tau/p-tau and Aβ_42_ (Henjum et al., 2018). The presence of sTREM2 in the CSF is associated with microglial activation, which supports the hypothesis that neuroinflammation plays a central role in the early stages of AD. This is further reinforced by the observation that sTREM2 concentrations increase following amyloid deposition and subsequent neuronal damage (Gispert et al., 2016; Suárez-Calvet et al., 2016a). However, it’s noteworthy that sTREM2 levels in the CSF do not consistently remain elevated across all AD stages.

Currently, the clinical significance of elevated serum sTREM2 levels in AD patients remains uncertain (Tanaka et al., 2019). While several studies have documented higher sTREM2 levels in the peripheral blood of AD patients compared to healthy counterparts (Hu et al., 2014; Casati et al., 2018), others have found no substantial differences (Piccio et al., 2008; Liu et al., 2018). This discrepancy could be attributed to various factors, including differences in patient demographics like gender, age, and disease stage. The origin of serum sTREM2 is also under investigation (Fahrenhold et al., 2018), with sources such as monocyte-derived dendritic cells expressing high levels of membrane-bound TREM2, which are then cleaved by sheddases (Song et al., 2017). The potential contribution of other cells, such as osteoclasts, to the circulating sTREM2 pool complicates the understanding of its impact on AD development, necessitating further study.

## 4 Regulatory mechanisms of sTREM2 shedding and metabolic degradation

The release of sTREM2 from its membrane-bound precursor is influenced by various stimuli, including LPS, interleukin-1β (IL-1β), oligomeric Aβ, Vascular Endothelial Growth Factor (VEGF), viral infections like HIV, and cytokines such as IL-13 and IL-4, which promote sTREM2 secretion (Wu et al., 2015; Gisslén et al., 2019; Zhong et al., 2019; Vilalta et al., 2021). Notably, LPS stimulation has been shown to decrease TREM2 surface expression and increase sTREM2 release in RAW264.7 cells, highlighting its role in TREM2 shedding and sTREM2 generation (Bandow et al., 2022). Additionally, BRI2 modulates sTREM2 functionality by inhibiting α-secretase-mediated cleavage of TREM2, affecting microglial phagocytic capabilities under pathological conditions (Yin T. et al., 2024). VEGF is recognized as a pivotal regulator of microglial phagocytosis in AD, facilitating the cleavage and release of sTREM2, thus enhancing the clearance of Aβ oligomers (de Gea et al., 2023). Studies report an age-related increase in CSF sTREM2 levels, suggesting rapid, demand-driven conversion of TREM2 to sTREM2, reflecting a regulatory need or a functional necessity for sTREM2, with the full-length protein serving as its precursor (Henjum et al., 2016).

The degradation pathways and body elimination processes for sTREM2 are not fully defined. Macrophages efficiently ingest sTREM2 (Wu et al., 2015), and sTREM2 introduced into the mouse brain is cleared within 3 days (Zhong et al., 2019). The role of meprin β in sTREM2 metabolism in the brain is under investigation, particularly its capacity to degrade sTREM2 following ADAM10/17-mediated inflammation which releases soluble meprin β (Berner et al., 2020). Moreover, sTREM2 variants from TREM2 gene splicing, particularly isoforms ENST00000373122 and ENST00000338469, add complexity to the sTREM2 pool, necessitating advanced analytical techniques to differentiate these isoforms in CSF (Piccio et al., 2016; Rauchmann et al., 2020). This underscores the importance of developing isoform-specific antibodies to better understand their roles in disease mechanisms. Ongoing research is essential to elucidate the functionalities of these distinct C-terminal sequences.

In conclusion, sTREM2 production results from both proteolytic cleavage and alternative splicing of TREM2. It actively participates in innate immune responses within the extracellular space. Genetic variations in *TREM2* influence sTREM2 levels, leading to considerable differences in sTREM2 concentrations in various conditions, such as AD. The mechanisms for sTREM2 breakdown and removal are still elusive, emphasizing the need for further investigation into the specific roles and implications of various sTREM2 forms in neurodegenerative diseases.

## 5 Modulators of sTREM2 levels: genetic, demographic, and environmental impacts

Research underscores the critical role of specific genetic loci and demographic variables in modulating levels of sTREM2 within the CSF. Key findings highlight a crucial genomic region on chromosome 11, adjacent to the *MS4A* gene cluster, significantly affecting CSF sTREM2 concentrations. Variants such as rs1582763, *MS4A6A* (p.A112T), and *MS4A4A* (p.M178V) have been found to elevate sTREM2 levels (Wang et al., 2024) whereas rs6591561 diminishes them (Winfree et al., 2024), and is also linked to an increased risk of AD (Piccio et al., 2016; Deming et al., 2019; Ferkingstad et al., 2021). This locus is not only expressed in microglia but also interacts functionally with TREM2, influencing microglial activation and predisposition to AD. Although this locus is pivotal, it shows no significant associations with other well-known AD risk factors like APOE and CD33 (Kamboh et al., 2012; Deming et al., 2019; Griciuc and Tanzi, 2021). Further research reveals that the rs2062323T allele in the *TREM1* gene is associated with increased CSF sTREM2 levels (Wang et al., 2023).

Demographic factors—particularly age, sex, and ethnicity—also significantly influence sTREM2 dynamics. Aging is consistently linked to increased CSF sTREM2 levels, suggesting its potential as a biomarker for age-related microglial activation and neuroinflammatory responses (Henjum et al., 2016; Piccio et al., 2016; Suárez-Calvet et al., 2016a,b, 2019; Gisslén et al., 2019; Knapskog et al., 2020; Moore et al., 2021). This elevation correlates with other markers of astrocyte activation and synaptic injury, highlighting the intricate interplay between microglial activity and neurodegenerative processes during aging (Moore et al., 2021). A recent study demonstrated elevated sTREM2 levels in the serum of long-COVID patients with cognitive impairment compared to healthy controls (Besteher et al., 2024), suggesting a shift in microglial reactivity in response to neuronal degeneration. While some studies report higher sTREM2 levels in males compared to females, these findings are not consistent across all cohorts (Piccio et al., 2016; Suárez-Calvet et al., 2016a,b; Knapskog et al., 2020; Wilson et al., 2020). Additionally, African American populations exhibit lower sTREM2 levels than Non-Hispanic Whites, paralleling lower levels of other AD biomarkers, which may reflect different genetic predispositions toward sTREM2 dynamics (Schindler et al., 2021). Lifestyle factors like physical exercise may influence sTREM2 levels and microglial function, though more research is needed in this area (Schindler et al., 2021). Furthermore, while CSF sTREM2 concentrations are thought to primarily reflect intrathecal production without correlation to peripheral blood levels or blood–brain barrier integrity, some findings suggest a negative correlation between CSF and plasma sTREM2 levels, associating higher plasma levels with aging (Piccio et al., 2008, 2016; Park et al., 2015). Epidemiological evidence also links prolonged exposure to fine particulate matter (PM2.5) with reduced CSF sTREM2 levels, suggesting impaired microglial function and increased neuroinflammation, potentially exacerbating AD pathology (Li et al., 2022). Additionally, high ferritin levels, indicative of increased brain iron, have been associated with elevated sTREM2 levels, particularly in individuals with higher baseline ferritin (Shi et al., 2022).

This body of evidence collectively illuminates the complex web of genetic, demographic, and potentially modifiable factors shaping sTREM2 levels, offering valuable insights into AD’s pathophysiology and highlighting the need for tailored approaches in studying sTREM2 across different neurological conditions.

## 6 sTREM2 dynamics and Alzheimer’s disease progression

**Figure 2:**
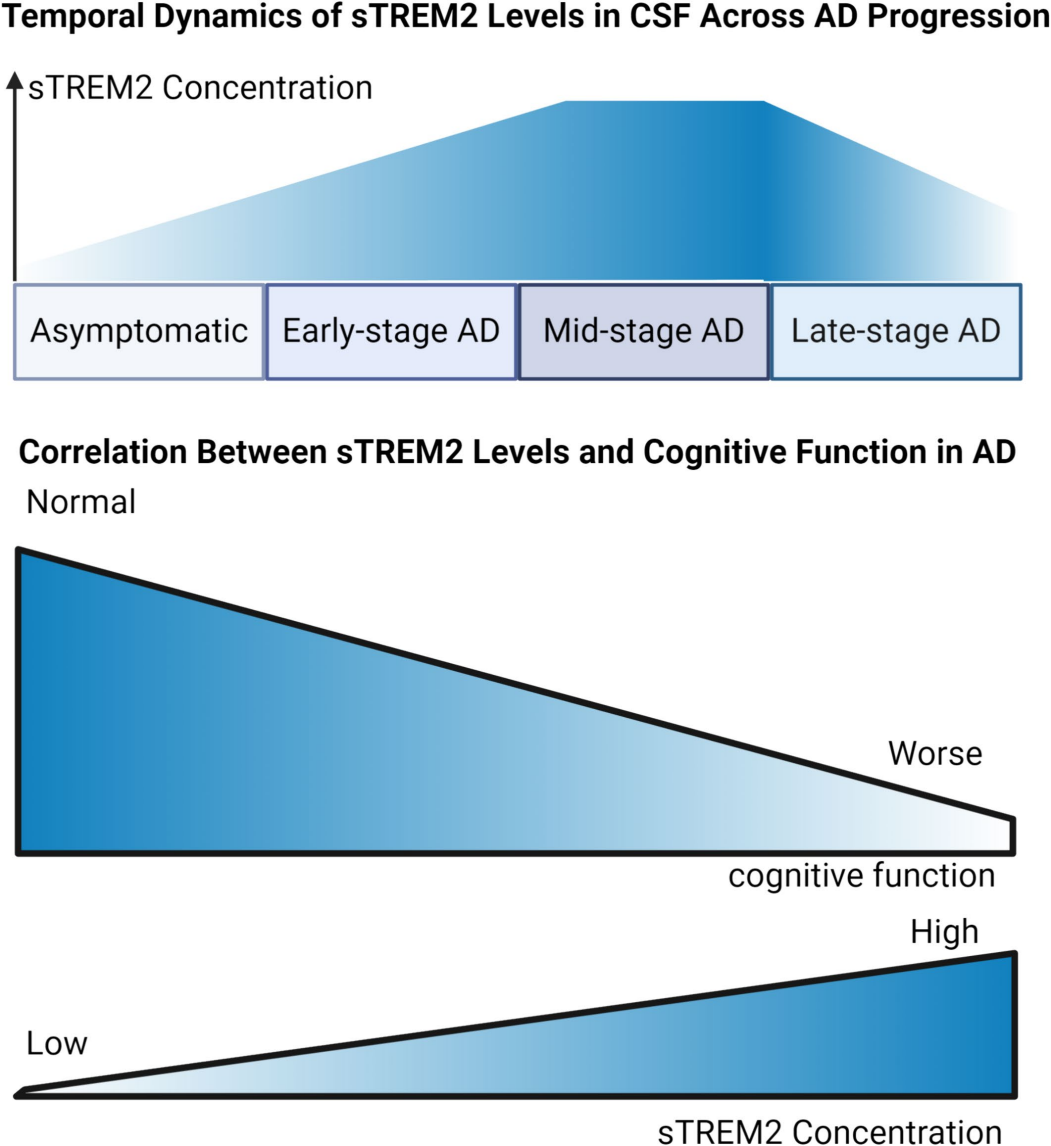
Dynamic Trends of sTREM2 Levels in Alzheimer’s Disease Progression. Temporal Dynamic of sTREM2 Levels in CSF Across AD Progression: Illustrating the trend of sTREM2 levels, the graph depicts an ascending trajectory against the stages of AD from asymptomatic to late-stage. It suggests an initial increase in sTREM2 concentration, which peaks during the early symptomatic phases, corresponding to heightened microglial activation, followed by a plateau or potential decrease in the late stages of AD. Correlation Between sTREM2 Levels and Cognitive Function in AD: Serving as a focused examination of the sTREM2-MMSE correlation, this graph presents a direct association between increased sTREM2 levels and changes in MMSE scores. It highlights how elevated sTREM2 levels are potentially linked with cognitive stability or decline, thereby reflecting their significance as a biomarker within the AD diagnostic spectrum.

Research into sTREM2 levels in the blood and CSF of individuals with AD presents a diverse range of findings. While the majority of cross-sectional studies observed a moderate increase in CSF sTREM2 levels among AD patients compared to cognitively normal controls (Heslegrave et al., 2016; Piccio et al., 2016; Ewers et al., 2019), there are reports of reduced (Kleinberger et al., 2014) or unchanged levels (Henjum et al., 2016; Knapskog et al., 2020). This variability might stem from the differing stages of disease among participants, including the inclusion of individuals with preclinical AD in control groups (Figure 2). CSF sTREM2 has shown promise as an indicator of cognitive decline in Parkinson’s disease (PD) (Qin et al., 2022), especially in PD with mild cognitive impairment (PD-MCI), where baseline CSF levels were significantly higher in patients who exhibited cognitive deterioration over a two-year period compared to those who remained cognitively stable (Paolini Paoletti et al., 2023). Remarkably, elevated CSF sTREM2 levels can be detected 12 to 14 years before the estimated onset of symptoms, marking its potential as an early indicator of neuroinflammatory changes (Li et al., 2024). CSF sTREM2 concentrations exhibit a stage-dependent increase in AD (Figure 2), peaking during early symptomatic phases, which aligns with heightened microglial activation in response to neuronal damage (Hok-A-Hin et al., 2023). Insights from the Dominantly Inherited Alzheimer Network (DIAN) have shown that CSF sTREM2 levels are elevated in familial AD mutation carriers up to 5 years before and after symptom onset but not beyond this period (Suárez-Calvet et al., 2016a). This finding is corroborated by recent studies demonstrating significantly higher CSF sTREM2 levels in individuals with mild cognitive impairment due to AD, particularly in those testing positive for CSF AD biomarkers (Giannisis et al., 2023). These elevated levels of sTREM2 not only correlate with the severity of neurofibrillary degeneration and cognitive decline but also reflect increased inflammasome activation (Španić Popovački et al., 2023). Another study highlighted that both CSF and plasma sTREM2 levels serve as significant predictive biomarkers for progression from MCI to AD, with lower CSF TREM2 levels indicating an elevated risk of disease progression (Zhou W. et al., 2023). Lower CSF sTREM2 levels are also associated with increased depressive symptoms and enhanced amyloid deposition in the early stages of AD, especially in individuals lacking the *APOE* ε4 allele (Wang et al., 2022b). Interestingly, higher sTREM2 concentrations were noted in cognitively normal individuals suspected of non-AD pathology (tau positive, amyloid negative) (Wang et al., 2022a), suggesting that sTREM2 elevation might more accurately indicate neuronal injury associated with tau rather than amyloid pathology.

In addition to its potential as a diagnostic marker, sTREM2 levels may offer prognostic insights. A study found that higher baseline CSF sTREM2 levels were linked to less severe declines in memory and cognitive functions over an 11-year span in individuals with AD, implying a protective role of microglial activation in the early stages of the disease (Ewers et al., 2019). Moreover, a higher CSF sTREM2 to p-tau181 ratio predicted slower progression from MCI to AD dementia or to more advanced symptomatic stages. Recent findings also revealed increased sTREM2 levels in the serum of long-COVID patients with cognitive impairment when compared to healthy controls (Besteher et al., 2024), suggesting a corresponding shift in microglial reactivity in response to neuronal degeneration. In patients whose diabetes control deteriorated, those with higher baseline levels of sTREM2 experienced a more pronounced decline in Mini-Mental State Examination (MMSE) scores over a 2-year period, which suggests a potential link between higher sTREM2 levels and the acceleration of cognitive impairment under conditions of poor glycemic control (Tanaka et al., 2022). This aligns with previous studies demonstrating an association between sTREM2 levels and neuronal injury markers in the early stages of AD.

Collectively, these findings enhance our understanding of sTREM2’s involvement in AD and MCI, highlighting its potential both as a biomarker of disease stage and as an indicator of microglial neuroprotective effects in the early stages of Alzheimer’s pathology.

## 7 Functional insights into sTREM2 in Alzheimer’s disease

### 7.1 The impact of sTREM2 shedding on TREM2 receptor signaling

The precise mechanisms through which sTREM2 shedding influences TREM2 receptor signaling and the inherent functions of sTREM2 remain somewhat enigmatic. It has been observed that stabilizing TREM2 on the cellular membrane with antibodies to hinder its proteolytic shedding by sTREM2 enhances not only downstream TREM2 signaling but also critical functions of the receptor, such as the phagocytic activity of microglial cells (Schlepckow et al., 2020; Szykowska et al., 2021). This observation suggests that the detachment of TREM2 from the membrane, facilitated by ADAM metalloprotease activity leading to sTREM2 production, might play a regulatory role in attenuating TREM2 receptor signaling. Genetic variant rs7922621 within a microglia-specific cis-regulatory element significantly impacts sTREM2 levels by modulating tetraspanin 14 expression, thereby influencing ADAM10 activity and the inflammatory response in AD (Yang et al., 2023), further supporting the idea that sTREM2 shedding may serve to modulate receptor activity. In line with this concept, an increase in both TREM2 protein levels and sTREM2 concentrations in the CSF has been associated with heightened microglial activation, as observed in inflammatory neurological conditions (Piccio et al., 2008; Diaz-Lucena et al., 2021), indicating elevated receptor signaling. It is posited that sTREM2 shedding may naturally follow ligand engagement, serving to regulate receptor activity by intercepting downstream signaling pathways. Consequently, the presence of sTREM2 in CSF or plasma could serve as an indicator of the extent of TREM2’s interaction with its ligand, including the dynamics of its shedding and the turnover of newly synthesized TREM2 within the cell, which is necessary for sustained receptor function. However, the precise dynamics between ligand engagement and the shedding process require further elucidation.

### 7.2 Ligand interactions with TREM2 and sTREM2: binding dynamics and functional consequences

The complexity of TREM2 ligands and their interactions with the receptor adds layers to our understanding of sTREM2’s functionality. TREM2 is recognized for its versatility, capable of binding a wide array of molecules with polyanionic traits, including bacterial membranes, myelin, lipoproteins such as ApoE, Aβ, DNA, and others (Poliani et al., 2015; Wang et al., 2015; Yeh et al., 2016; Cignarella et al., 2020), suggesting that TREM2’s activation is highly context-dependent. Notably, TREM2’s ability to bind to membrane-associated ligands in-trans on macrophages (Hamerman et al., 2006) and astrocytes (Piccio et al., 2007), with emerging evidence of sTREM2 interactions with neuronal surfaces (Song et al., 2018; Zhong et al., 2019), points to a multifaceted role in cellular communication and immune response. However, the extent to which sTREM2, like TREM2, can engage in-trans ligands present on the surface of neighboring cells remains a question that warrants further investigation.

TREM2’s pivotal role in Aβ phagocytosis within the brain is underscored by the specific interaction between the TREM2 ectodomain and Aβ fibrils, facilitated by the V-type immunoglobulin-like domain (Zhong et al., 2018). Modifications such as glycosylation and sulfidation further refine TREM2’s ligand affinity (Kober et al., 2016), enhancing its functional capacity. Among TREM2’s ligands, lipoprotein particles and apolipoproteins, notably Low-Density Lipoprotein (LDL), Clusterin (CLU)/APOJ, and APOE, have been thoroughly investigated (Daws et al., 2003; Yeh et al., 2016; Nugent et al., 2020). Studies reveal that APOE, particularly its ε4 allele—a known risk factor for late-onset AD (Nicolas, 2024)-acts as an agonist in this interaction, affecting AD pathology through the TREM2-APOE complex (Bailey et al., 2015; Jendresen et al., 2017). This interaction is significant, with specific amino acid sequences in APOE identified as critical for binding to TREM2’s ectodomain (Yeh et al., 2016; Zhao et al., 2018), promoting the clearance of apoptotic neurons and misfolded proteins via the TREM2 pathway (Atagi et al., 2015). Furthermore, Aβ’s interaction with APOE’s lipid-binding region activates TREM2, contributing to the amyloid plaque-associated TREM2 activation. This evidence collectively suggests a synergistic relationship between TREM2 and APOE in modulating AD progression, highlighting their collaborative role in Aβ uptake and clearance in the context of the disease. In a pivotal study exploring the role of sTREM2 in post-stroke recovery, sTREM2 was identified as a negative regulator of erythrophagocytosis through CD36 receptor recycling, suggesting that modulation of sTREM2 activity could enhance hematoma resolution and neurorehabilitation outcomes following intracerebral hemorrhage (Zhou H. et al., 2023). These findings underscore the multifaceted nature of sTREM2’s interactions and its potential as a therapeutic target in various neurological conditions.

### 7.3 sTREM2 and Aβ: modulating amyloid aggregation and neurotoxicity

sTREM2 exhibits specificity in binding to oligomeric forms of Aβ, with significantly less affinity for monomeric or fibrillar structures (Lessard et al., 2018; Zhao et al., 2018; Zhong et al., 2018; Vilalta et al., 2021). Research reveals that the TREM2 ectodomain’s affinity varies across different amyloid structures, with Aβ oligomers demonstrating the highest binding affinity, regardless of whether they are Aβ_42_ monomers, Aβ_42_ fibrils, or Aβ_40_ monomers (Lessard et al., 2018). Notably, the interaction between sTREM2 and Aβ oligomers is characterized by a slow dissociation rate, effectively hindering Aβ oligomers from binding to other potential ligands. Further investigation uncovered that sTREM2 inhibits both the oligomerization and fibrillization of Aβ at specific molar ratios (1 sTREM2 to 100 Aβ), and higher concentrations of sTREM2 can disaggregate Aβ oligomers and fibrils, suggesting sTREM2’s role as an extracellular chaperone (Vilalta et al., 2021). This function allows sTREM2 to prevent Aβ from forming neurotoxic aggregates and even revert such aggregates back to less harmful forms, thus attenuating Aβ’s neurotoxic effects (Kober et al., 2020; Vilalta et al., 2021). Conversely, the R47H variant of sTREM2 demonstrates diminished binding to Aβ oligomers, paradoxically enhancing the aggregation of Aβ into protofibrils and exacerbating neuronal loss *in vitro* (Vilalta et al., 2021). This suggests that the R47H mutation might not only diminish sTREM2’s neuroprotective abilities but could also contribute to Aβ’s neurotoxicity by promoting its aggregation into more harmful structures. Administering recombinant sTREM2 has been shown to reduce Aβ accumulation and ameliorate behavioral impairments in the 5 × FAD mouse model of AD (Zhong et al., 2019), underscoring sTREM2’s potential in mitigating Aβ-driven pathology in AD. These findings collectively highlight sTREM2’s therapeutic value in targeting neurodegenerative disorders, offering insights into its ability to modulate Aβ aggregation and attenuate its neurotoxic effects. As research continues to unravel the intricacies of sTREM2’s interactions with Aβ, it is becoming increasingly evident that harnessing sTREM2’s unique properties could pave the way for novel therapeutic strategies in the fight against AD and other neurodegenerative diseases.

### 7.4 Evaluating sTREM2: effects on amyloid pathology in transgenic animal models

Injecting sTREM2 into the brains of mice engineered to express amyloid precursor protein (APP) led to a notable reduction in amyloid plaque burden (Zhong et al., 2019). Moreover, employing viral vectors to express sTREM2 in these APP mice diminished plaque loads and significantly reversed spatial memory deficits and impairments in long-term potentiation (LTP) (Moutinho et al., 2023). These observations underscore sTREM2’s role in guarding against amyloid pathology, potentially through mechanisms that involve altering Aβ aggregation dynamics or enhancing microglial activity to engulf plaques. Interestingly, specific sTREM2 segments (amino acids 51–81) activated microglia without binding Aβ or tangibly affecting amyloid pathology, whereas a slightly larger fragment (amino acids 41–81) demonstrated the capability to both bind Aβ and significantly reduce amyloid deposition, outperforming the full-length protein (Sheng et al., 2021). Further, sTREM2 influences microglial morphology and sustains activation by delivering Fc-TREM2 to the hippocampus in both wild-type and Trem2 knockout mice (Zhong et al., 2017). This indicates a primary protective mechanism of sTREM2 against amyloid pathology through its interaction with Aβ.

In contrast, TREM2-deficient mice, crossed with APP mice, exhibited plaques that were more fibrous and less compact (Condello et al., 2015; Wang et al., 2016; Yuan et al., 2016; Song et al., 2018), a phenomenon previously attributed to diminished microglial plaque phagocytosis due to the absence of full-length TREM2. Alternatively, this could result from an impaired ability of sTREM2 to hinder Aβ aggregation or its reduced capacity to stimulate microglial plaque clearance. Upon the formation of the TREM2-Aβ complex, microglia are spurred into action, embarking on targeted phagocytosis to clear aggregated proteins while concurrently preventing the spread of plaques to adjacent healthy tissue. This is complemented by the activation of microglia around plaques to release inflammatory mediators and kick-start anti-plaque signaling mechanisms. Further, TREM2-deficient models show an increase in Aβ seeding (Parhizkar et al., 2019), potentially due to a decrease in microglial phagocytosis of Aβ seeds, a process facilitated by full-length TREM2, or possibly due to a lack of Aβ aggregation blockade by sTREM2. In models expressing human wild-type TREM2, sTREM2 has been observed in close association with amyloid plaques (Song et al., 2018), reinforcing its regulatory role in plaque management. Notably, both sTREM2 and its full-length counterpart share the ability to bind Aβ oligomers, though the comparative efficacy of these interactions in plaque regulation remains to be explored (Vilalta et al., 2021). Furthermore, individuals and mice harboring the R47H TREM2 variant exhibit plaques with increased fibrousness and neuritic pathology (Yuan et al., 2016), which could be explained by either the variant sTREM2 fostering Aβ fibrillation or a diminished microglial response to plaque phagocytosis. This nuanced understanding of sTREM2’s interplay with amyloid pathology not only elucidates its protective mechanisms but also opens avenues for therapeutic interventions targeting AD.

### 7.5 TREM2 and sTREM2: central players in neuroinflammation and tau pathology

AD is widely recognized not merely as a disorder of abnormal protein accumulation but also as one deeply characterized by extensive neuroinflammation (Liu et al., 2023; Cohen et al., 2024). Fundamental to understanding AD’s complexity is the role of TREM2, a key regulator in the immune response associated with this disease. Genetic studies have robustly demonstrated that TREM2 is instrumental in managing neuroinflammatory processes (Nicolas, 2024), and its activity is crucial even before the deposition of Aβ (Shen et al., 2024). The critical function of TREM2 begins with its ectodomain binding to a specific ligand, which triggers a sequence of events starting with proteolytic cleavage (Wunderlich et al., 2013). This cleavage allows the intracellular fragment of TREM2 to interact with DAP12, subsequently activating the Syk–PI3K/MAPK signaling pathway. This activation is essential as it promotes the release of IP^3−^gated Ca^2+^ stores that are vital for phagocytosis (Sasaki et al., 2015; Glebov et al., 2016). sTREM2 emerges as a significant modulator in this process. It plays a crucial role in microglial activation and significantly influences cytokine expression in THP-1 monocytes and macrophages through the MAPK–JNK signaling pathway. This modulation helps initiate pro-inflammatory responses at the early stages of AD and fosters anti-inflammatory activities in the later stages (Rauchmann et al., 2020; Pascoal et al., 2021; Arsenault et al., 2024). Moreover, alterations in peripheral sTREM2-related inflammatory markers such as Fibroblast Growth Factor 2 (FGF-2), Granulocyte-Macrophage Colony-Stimulating Factor (GM-CSF), and IL-1β significantly correlate with stages of AD, reflecting sTREM2 levels (Weber et al., 2022). In primary microglia from both wild-type and Trem2-knockout mice models, sTREM2 stimulates the production of inflammatory cytokines through the NF-κB pathway and promotes microglial survival via the Akt/GSK3β/β-catenin signaling pathway (Zhong et al., 2017, 2019). The diversity in microglial responses further complicates the immune landscape in AD. Microglia exhibit heterogeneity with M1 and M2 phenotypes, where M1 microglia are pro-inflammatory and cytotoxic, while M2 microglia provide anti-inflammatory and neuroprotective functions (Li and Zhang, 2018). Notably, overexpressing TREM2 in P301S tau transgenic mice has been shown to activate M2 microglia, which leads to a reduction in the release of pro-inflammatory cytokines, demonstrating TREM2’s therapeutic potential (Neumann and Takahashi, 2007; Jiang et al., 2016). Furthermore, the levels of sTREM2 in CSF serve as a dynamic biomarker for AD progression, reflecting microglial states and their engagement in clearing amyloid plaques and regulating inflammation (Kuchenbecker et al., 2024; Yin S. et al., 2024). This is particularly notable during the early symptomatic stages of AD. Additionally, studies have identified a close link between sTREM2 levels and specific CSF metabolites such as stearoyl sphingomyelin and palmitoyl sphingomyelin, which are involved in cellular signaling and potentially influence microglial activation (Dong et al., 2023). However, the role of TREM2 in AD presents a complex paradox. While TREM2’s activation generally supports anti-inflammatory processes, its deficiency or loss of function has been found to reduce neurodegeneration and mitigate tau pathology in some models, suggesting that its exact role may vary significantly depending on the pathological context (Leyns et al., 2017; Jiang et al., 2018; Sayed et al., 2018). Furthermore, research has shown that elevated sTREM2 levels correlate with increased phosphorylated tau, enhanced glucose metabolism, and decreased tau propagation in brain networks, highlighting sTREM2’s impact on both tau pathology and neuronal connectivity (Biel et al., 2023; Nabizadeh, 2023). Complex interactions continue to be explored, including how TREM2 and sTREM2 correlate with tau and phosphorylated tau (p-tau) levels in both CSF samples and post-mortem AD brain tissues, unaffected by the presence of Aβ (Cruchaga et al., 2013; Suárez-Calvet et al., 2019; Vogels et al., 2019). These findings suggest that TREM2 and sTREM2’s roles extend beyond simple inflammatory modulation to significant implications in tauopathy and overall disease progression. The roles of TREM2 and sTREM2 are further highlighted in other contexts such as long-COVID, where cognitive impairments associated with elevated IFNγ and IL-10 levels suggest a sustained inflammatory state that may perpetuate tissue damage and symptom severity (Besteher et al., 2024). Additionally, interactions with neuronal transgelin-2 (TG2) indicate novel pathways for mitigating tau phosphorylation and cognitive deficits, presenting new therapeutic possibilities for addressing neurodegeneration (Zhang et al., 2023b).

### 7.6 Synaptic roles of TREM2 and sTREM2: pruning and connectivity in the brain

TREM2 and sTREM2 are hypothesized to play a crucial role in synaptic functions within the brain. It’s suggested that TREM2’s ectodomain, or sTREM2, acts as an extracellular signal that binds to synaptic surface receptors, thereby guiding microglia during the synaptic pruning necessary for proper brain connectivity (Filipello et al., 2018). This involvement is pivotal in maintaining inter-neuronal signal transmission. However, the precise mechanisms by which TREM2 influences human synapses remain to be fully elucidated. Studies in AD mouse models have linked TREM2 deficiency with impairments in synaptic structure and cognitive decline, indicating that proper synaptic pruning, which is essential for normal cognition, is compromised under TREM2 deficiency. This leads to synaptic and axonal dystrophy, and a reduction in hippocampal microglia, which are crucial for synaptic pruning (Kajiwara et al., 2016; Bohlen et al., 2019; Jay et al., 2019; Sheng et al., 2019). Furthermore, TREM2 interacts with complement proteins such as C1q and C3, playing a significant role in the elimination of inappropriate synaptic connections during the maturation of neural circuits (Schafer et al., 2012; Stephan et al., 2012). Research by Woo et al. demonstrated that sTREM2 mediates synaptic loss independently before tau accumulation becomes evident. However, in later stages of the disease, when tau accumulation is visible, astrogliosis also contributes to synaptic damage (Woo et al., 2023). Moreover, elevated levels of sTREM2 in the CSF have been independently associated with presynaptic loss, as evidenced by increased Growth Associated Protein 43 (GAP-43) levels, suggesting that sTREM2-mediated microglial reactivity might drive synaptic dysfunction independently of β-amyloid and tau pathologies (Lan et al., 2024). Additionally, concentrations of various forms of sTREM2, reported in the CSF of AD patients, have been found to inhibit LTP independently of amyloid plaque presence and related pathology. Intriguingly, blocking Gamma-Aminobutyric Acid (GABA) receptors prevented the LTP-impairing actions of sTREM2, suggesting a dependency on the activation of these receptors, or that the removal of tonic GABA transmission could counteract the inhibitory impact of sTREM2 on neuronal excitability (Moutinho et al., 2023).

## 8 Concluding perspectives and future directions in TREM2 and sTREM2 research

The extensive evidence presented in this review underscores the pivotal roles of TREM2 and sTREM2 in the complex pathogenesis of AD. Their involvement in microglial function, neuroinflammation, and the modulation of amyloid and tau pathologies highlights their potential as therapeutic targets and biomarkers for disease progression. However, despite the significant progress made in understanding their functions, several key aspects of TREM2 and sTREM2 biology remain to be elucidated. In this concluding section, we summarize the main findings, discuss the challenges and opportunities, and outline the critical directions for future research in this field.

### 8.1 Overview of findings

TREM2, through its extracellular domain, binds to a variety of ligands, orchestrating a myriad of functions within the brain. These interactions not only sequester harmful substances but also activate the DAP12–Syk signaling pathway, potentially leading to an inflammatory response. Additionally, sTREM2 has the capacity to directly engage with pathogenic entities like Aβ or indirectly modulate inflammatory processes (Song et al., 2018; Karanfilian et al., 2020), even independently causes synaptic cleavage before tau accumulation appears. These insights underscore the pivotal roles of TREM2’s extracellular domain and sTREM2 in the evolution of AD, positioning them as promising targets for therapeutic intervention. However, the interplay between protein aggregation and neuroinflammation presents conflicting data (Mosley, 2015; Labzin et al., 2018), with some studies suggesting that misfolded Aβ fibrils disrupt brain homeostasis, while others argue that protein accumulation triggers inflammation. The initiating factor of AD—be it protein deposition or disturbed homeostasis—remains uncertain, though the involvement of the innate immune system is clear (Castro-Gomez and Heneka, 2024).

Elevated serum levels of sTREM2 may also serve as a promising biomarker for identifying mild cognitive impairment in patients with obstructive sleep apnea, correlating with lower cognitive performance as assessed by the Montreal Cognitive Assessment (Jiahuan et al., 2022). These findings advocate for sTREM2’s role in mediating microglial-associated inflammatory responses and suggest its potential as a protective agent in AD. Nonetheless, the regulatory effects of TREM2 on immune responses remain contentious, with some studies indicating that TREM2 may incite microglia to release neurotoxic pro-inflammatory cytokines, such as IL-1β, TNFα, and IL-6 (Nordengen et al., 2019). To reconcile these divergent results, an understanding of AD’s progression and TREM2’s impact on resident microglia is essential (Zhang et al., 2023a). At early and mid-stages of AD, TREM2 appears protective, but this role may pivot to a detrimental one by activating the adaptive immune system in the disease’s later phases (Suárez-Calvet et al., 2016b; Jay et al., 2017), reflecting the dynamic activation states of microglia throughout AD. sTREM2, acting as a decoy receptor, can induce the production of inflammatory cytokines, prompting microglial transformation and immune responses to counteract early-stage AD threats (Karanfilian et al., 2020).

In summary, TREM2 and sTREM2’s roles in AD pathogenesis are complex, involving ligand interactions, microglial function regulation, neuroinflammation modulation, and direct impacts on amyloid and tau pathologies. While evidence predominantly supports TREM2/sTREM2’s neuroprotective function in AD’s early stages, conflicting observations and unanswered questions about their actions, especially in later disease stages, persist. Future research is essential to clarify TREM2/sTREM2’s nuanced roles across AD’s spectrum and to verify their therapeutic viability.

### 8.2 Progress in the development of TREM2 and sTREM2-related drugs

Several companies had identified antibodies targeting the ectodomain of TREM2, which likely stimulate TREM2 signaling via crosslinking (Cheng et al., 2018; Cignarella et al., 2020; Price et al., 2020). These antibodies, such as AL002a/c developed by Alector, bind to the extracellular domain of TREM2 and activate downstream signaling pathways, including phosphorylation of DAP12 and Syk. In preclinical studies, these agonistic antibodies have been shown to enhance microglial survival, proliferation, and phagocytosis of myelin debris and Aβ.

Another approach involves preventing the shedding of the TREM2 extracellular domain by ADAM proteases using antibodies that bind to the stalk region near the cleavage site, such as clone 4D9 (Schlepckow et al., 2020). However, more work is required to identify a “therapeutic molecular signature” and functional outcomes stimulated by TREM2 agonistic antibodies. Additionally, it remains to be demonstrated whether CSF concentrations of sTREM2 can be used as a therapeutic biomarker, as recently proposed (Wang et al., 2020).

Several TREM2 agonists were in clinical. These included Denali’s DNL919 and Alector’s AL002 antibodies, which were being evaluated in Phase 1 and Phase 2 trials, respectively, though neither reported results at the AD/PD conference that year. Vigil Neuroscience, a company based in Watertown, Massachusetts, was evaluating its TREM2 agonist antibody, VGL101, in people with ALSP, a rare genetic disorder caused by a mutation in the CSF-1R gene. However, for AD—a much larger indication that requires chronic dosing—Vigil took a small-molecule approach. The company had originally acquired its TREM2 agonist antibody and a suite of TREM2 small-molecule agonists from Amgen in 2020, after Amgen discontinued its neuroscience programs. At the 2023 AD/PD conference, Vigil’s Christian Mirescu presented preclinical data on the company’s lead TREM2 agonists, which had been selected and optimized for brain permeability, oral availability, and potency. Mirescu claimed they worked like “molecular glue, “promoting the clustering of TREM2 receptors on the microglial cell surface. In cultured human microglia, this clustering boosted TREM2 signaling and reduced shedding. Vigil aimed to file an IND application for its lead TREM2 agonist. Before testing the agonists in the broader AD population, the company planned to evaluate them in people who carry loss-of-function mutations in TREM2 or other microglial genes.

### 8.3 Directions for future research

1. Development of Advanced Imaging Tools: There is a critical need for non-invasive imaging technologies capable of monitoring TREM2 and sTREM2 in living patients. Techniques such as PET imaging tagged to specific TREM2/sTREM2 antibodies could allow real-time observation of their distribution and activity, facilitating a better understanding of their roles throughout the progression of AD.
2. Longitudinal Cohort Studies: Implementing longitudinal studies that track TREM2 and sTREM2 levels alongside cognitive decline and other biomarkers of AD could clarify their roles as potential predictive or diagnostic tools. Such studies should aim to include a diverse participant pool to assess the impact of genetic backgrounds and environmental exposures on TREM2/sTREM2 dynamics.
3. Integrated Systems Biology Approaches: Utilizing systems biology to integrate data from genomics, proteomics, and metabolomics could help elucidate the complex interactions and regulatory networks involving TREM2 and sTREM2. This approach would also aid in identifying potential therapeutic targets within these pathways.
4. Clinical Trials Targeting TREM2/sTREM2 Pathways: Despite the theoretical benefits of modulating TREM2 and sTREM2 activity, fundamental research based on clinical studies and non-interventional approaches is necessary to evaluate the potential efficacy and safety of targeting TREM2/sTREM2 pathways. These studies should be designed to not only assess potential therapeutic outcomes but also to monitor any unintended effects on immune function within the brain and the circulatory system, given the roles of TREM2 and sTREM2 in neuroinflammation and systemic immune responses.

## Author contributions

CL: Writing – original draft. YK: Writing – original draft. QC: Writing – original draft. JZ: Writing – original draft. XP: Writing – review & editing. JM: Writing – review & editing.
